# Hypoxia promotes colorectal cancer cell migration and invasion in a SIRT1-dependent manner

**DOI:** 10.1186/s12935-019-0819-9

**Published:** 2019-04-30

**Authors:** Shentong Yu, Ru Zhou, Tong Yang, Shuang Liu, Zhuqing Cui, Qing Qiao, Jing Zhang

**Affiliations:** 1 State Key Laboratory of Cancer Biology, Department of Pathology, Xijing Hospital, The Fourth Military Medical University, Xi’an, 710032 China; 2 School of Basic Medicine, The Fourth Military Medical University, Xi’an, 710032 China; 3 Department of General Surgery, Tangdu Hospital, The Fourth Military Medical University, Xi’an, 710038 Shaanxi China

**Keywords:** Hypoxia, SIRT1, EGR1, Colorectal cancer, Migration, Invasion

## Abstract

**Background:**

Hypoxic microenvironments play a significant role in the progression of colorectal cancer (CRC). Silencing information regulator 1 (SIRT1), a class III histone deacetylase, modulates the multiple biological behaviors of cancer. However, its role in CRC remains unclear. This study aims to explore the role of SIRT1 in CRC migration and invasion under hypoxia.

**Methods:**

SIRT1 protein and mRNA levels were detected by Western blotting and real-time PCR in CRC cells exposed to hypoxia (1% O_2_). The migration and invasion abilities of SW480 and HCT116 cells with SIRT1 overexpression or knockdown were studied with transwell assays, and the results were confirmed by those of treatment with specific SIRT1 activator (SRT1720) and inhibitor (EX527). The dual-luciferase reporter systems with a series of SIRT1 promoter truncations were used to analyze their transcriptional activities, respectively. After a bioinformatic analysis of potential transcription factors, the direct interaction between the transcription factor and SIRT1 promoter was determined by chromatin immunoprecipitation (ChIP) assays. Western blot and real-time PCR assays were used to detect the activation and acetylation levels of the NF-κB pathway.

**Results:**

The protein and mRNA levels of SIRT1 were significantly decreased under hypoxia, and these effects were replicated by cobalt chloride treatment. Hypoxia promoted cell migration and invasion, which were impeded by the overexpression or activation of SIRT1 and promoted by the knockdown or inhibition of SIRT1. The dual-luciferase reporter gene and ChIP analyses revealed that the core regulatory elements located 100 bp upstream of the SIRT1 promoter and early growth response factor 1 (EGR1) could interact with this DNA sequence. Subsequent rescue experiments suggested that EGR1 was essential for hypoxia-mediated SIRT1 transcriptional suppression. Western blot analyses demonstrated that SIRT1 overexpression eliminated the p65 acetylation induced by hypoxia along with the decreased MMP-2/-9, suggesting that NF-κB was a direct downstream target of SIRT1 and might regulate cell migration and invasion through MMP-2/-9.

**Conclusions:**

Our results establish for the first time that EGR1 plays an important role in regulating SIRT1 expression under hypoxia. Hypoxia promotes CRC cell migration and invasion in a SIRT1-dependent manner. And a potential SIRT1/NF-κB/MMP-2/-9 axis modulates this process.

**Electronic supplementary material:**

The online version of this article (10.1186/s12935-019-0819-9) contains supplementary material, which is available to authorized users.

## Background

Colorectal cancer (CRC) is the third most common cancer and one of the leading causes of cancer-related death worldwide [1]. What is worse, metastatic cases account for approximately 40% to 50% of newly diagnosed patients, whose overall survival (OS) reaches only up to 30 months [2]. Despite the fact that personalized medicine dramatically improves the outcome of cancer treatment, the prognosis of metastatic CRC remains poor. There is no doubt that a better molecular understanding of CRC will provide clinicians with more choices to achieve better outcomes for patients. However, our understanding of the underlying molecular mechanisms through which cancer migrates and metastasizes is not sufficient to address this problem. Some important biological behaviors have been discovered to partly illuminate this process.

With the rapid expansion of cancer cells, the central regions of most solid tumors are subjected to hypoxia because of inadequate vascularization, and the presence of a hypoxic microenvironment and the activation of downstream effectors are common features [3]. It is generally considered that hypoxia causes resistance to chemotherapy and initiates a number of events, such as epithelial-mesenchymal transition (EMT), inflammatory cell infiltration, and autophagy [4, 5]. The formation of the so-called “pro-metastatic niche” facilitates the progression of CRC to a large extent.

Silencing information regulator 1 (SIRT1) is a highly evolutionarily conserved histone and non-histone deacetylase. Altered SIRT1 expression is observed during ischemia/reperfusion, and SIRT1 exerts protective roles in the heart, liver and brain tissues [6, 7]. However, its dysregulation is also frequently observed in many tumor types, and it is thought to play multifaceted roles (promotor or inhibitor) in tumor progression. In ovarian cancer, hypoxia induces the transcriptional repression of SIRT1, which contributes to EMT and enhances cancer metastasis [8]. Conversely, under hypoxic conditions, SIRT1 is a direct downstream target of hypoxia-inducible factor 1α (HIF-1α), which orchestrates with NF-κB pathway to promote cancer stem cell-like properties in ovarian cancer cells [9]. From this perspective, alterations in SIRT1 expression under hypoxia and its roles in the hypoxic microenvironment need further research. This information may shed light on the mechanisms of CRC migration and invasion.

Early growth response factor 1 (EGR1) is a zinc-finger transcription factor that binds to canonical GC-rich motifs. EGR1 is induced by a variety of stimuli, including hypoxia, ionizing radiation, hyperglycemia and chemotherapy drugs, and it promotes or inhibits tumor proliferation [10]. EGR1 expression varies under hypoxic conditions in different cellular contexts and regulates diverse biological processes. In addition, there is evidence that EGR1 binds directly to the SIRT1 promoter and interacts with SIRT1 to regulate its function [11]. Hence, we wondered whether EGR1 regulates SIRT1 expression under hypoxic conditions and whether the hypoxia/EGR1/SIRT1 axis contributes to the progression of CRC.

In this paper, we report that EGR1 binds directly to the GC-rich box 100 bp upstream of the SIRT1 promoter and mediates hypoxia-induced SIRT1 transcriptional suppression in CRC cells. In addition, the hypoxia/EGR1/SIRT1 axis contributes to the migration and metastasis of CRC cells. Moreover, decreased SIRT1 activity promotes the acetylation of NF-κB and the activation of matrix metalloproteinase 2/9 (MMP-2/-9) and boosts the migration and invasion of CRC cells. As a consequence, the restoration of SIRT1 may suppress CRC progression and might be a promising target for hindering the development of CRC.

## Methods and materials

### Cell culture

HEK-293T, HCT116 and SW480 cell lines were purchased from the American Tissue Culture Collection (ATCC, Manassas, VA, USA). The HEK-293T cell line was cultured in DMEM with 10% FBS, the HCT116 cell line was cultured in McCoy’s 5A medium with 10% FBS, and the SW480 cell line was cultured in Leibovitz’s L-15 medium with 10% FBS. All cell lines were cultured at 37 °C in a humidified atmosphere containing 5% CO_2_. Hypoxia (1% O_2_) was maintained by pumping a mixture of gases (95% N_2_, 5% CO_2_) into a Billups-Rothenberg chamber (Del Mar, CA, USA) at 37 °C and monitored by a gas flowmeter.

### Lentivirus infection and plasmid transfection

Lentiviruses were used to generate cells with SIRT1 overexpression or downregulation. Based on the protein expression levels of SIRT1 in several CRC cell lines (Additional file 1: Figure S1A), HCT116 cells were stably transfected with an empty vector (Con077) or SIRT1-shRNA (GeneChem, Shanghai, China). SW480 cells were stably transfected with an empty vector (Con195) or lenti-SIRT1 (GeneChem, Shanghai, China). The transfected cells were then screened with puromycin (4 μg/mL) for several passages. SIRT1 promoter luciferase reporter plasmids (truncated SIRT1 promoter sequences and mutated 100 bp promoter sequences inserted into the GV354 vector, GeneChem, Shanghai, China) as well as EGR1, Sp1 and USF2 overexpression plasmids (constructed using GV230 and GV141 vectors, GeneChem, Shanghai, China) were transiently transfected into HEK-293T cells with Lipofectamine 2000 (Invitrogen, Carlsbad, CA, USA).

### RNA extraction and real-time PCR

Total RNA extraction was performed with RNAiso plus (TaKaRa, Shiga, Japan) according to the manufacturer’s instructions. Reverse transcription was then performed with PrimeScript™ RT Master Mix (TaKaRa, Shiga, Japan). Real-time PCR was performed on an Agilent Technologies system (Santa Clara, CA, USA) using TB Green Premix Ex Taq II (TaKaRa, Shiga, Japan) and the following primers:

**Table:** 

Primers	Sequences
SIRT1 forward	5′-GCAGATTAGTAGGCGGCTTG-3′
SIRT1 reverse	5′-TCATCCTCCATGGGTTCTTC-3′
β-Actin forward	5′-GGACTTCGAGCAAGAGATGG-3′
β-Actin reverse	5′-AGCACTGTGTTGGCGTACAG-3′
MMP-2 forward	5′-CTCTCCTGACATTGACCTTGGCAC-3′
MMP-2 reverse	5′-GTATTCATTCCCTGCAAAGAACAC-3′
MMP-9 forward	5′-AATCTCACCGACAGGCAGCT-3′
MMP-9 reverse	5′-CCAAACTGGATGACGATGTC-3′
TIMP-2 forward	5′-GTGGACTCTGGAAACGACAT-3′
TIMP-2 reverse	5′-CCAGGAAGGGATGTCAGAGC-3′
TIMP-3 forward	5′-TACCGAGGCTTCACCAAGATG-3′
TIMP-3 reverse	5′-CAGGACTTGATCTTGCAGTTAC-3′

Three duplicate wells were used for each sample, and each group was analyzed three times. Relative quantitative values were then calculated by the 2^−ΔΔCt^ method.

### Western blot analysis

Cell extracts were prepared by lysis in 1× SDS loading buffer; then, the samples were boiled and subjected to Western blot analysis. Proteins (30 μg) were transferred from gels onto PVDF membranes (Millipore, Billerica, MA, USA) and blocked in 5% skimmed milk for 2 h. The following antibodies were used in the experiment to detect proteins: rabbit anti-SIRT1 (1:1000, Abcam, Cambridge, UK), EGR1 (1:1000, Abcam), USF2 (1:1000, Abcam), NF-κB p65 (acetyl K310) (1:1000, Abcam), TIMP-2 (1:1000, Abcam), MMP-2 (1:500, ProteinTech, Wuhan, China), MMP-9 (1:500, ProteinTech), mouse anti-β-actin (1:2000, Cell Signaling Technologies, Beverly, MA, USA), and NF-κB p65 (1:100, Santa Cruz, Dallas, Texas, USA). The antibodies were incubated with the membranes at room temperature for 1 h and then at 4 °C overnight. HRP-linked anti-rabbit and anti-mouse secondary antibodies were incubated with the membranes at 4 °C for 1 h. All blotting was detected by the enhanced chemiluminescence (ECL) detection method.

### Dual-luciferase reporter assay

SIRT1 promoter region sequences were segmented from distal to proximal and fused with firefly and Renilla luciferase cDNA in a GV354 vector (GeneChem, Shanghai, China). All constructs were verified by sequencing. HEK-293T cells were seeded in 24 wells at a density of 5 × 10^4^ cells per well 24 h before transfection. These constructs were then transfected into cells and maintained at 37 °C for 12 h. Dual-luciferase reporter assays were performed in accordance with the manufacturer’s instructions (Promega, Madison, WI, USA), and firefly and Renilla luciferase activities were assessed using a dual-luciferase reporter assay system (Promega, Madison, WI, USA). Three duplicate wells were used for each sample, and each sample was analyzed three times. The ratio of firefly luciferase activity to Renilla luciferase activity was used to indicate the relative luciferase activity.

### Chromatin immunoprecipitation

Chromatin immunoprecipitation (ChIP) assays were performed using a SimpleChIP Plus Kit (Cell Signaling Technologies) according to the manufacturer’s instructions. HEK-293T cells were seeded in 10 cm dishes at a density of 2 × 10^6^ prior to the experiment. Cells were cross-linked with 1% formaldehyde, and the reaction was terminated with glycine. Chromatins were then digested by micrococcal nuclease. Antibodies against IgG (negative control, anti-rabbit monoclonal IgG, Cell Signaling Technologies) and EGR1 (Cell Signaling Technologies) and Protein G magnetic beads were used to immunoprecipitate the protein–nucleic acid complexes. Chromatin elution and purification were performed after that. The DNA sequence of the SIRT1 promoter bound to EGR1 was amplified by PCR, and the following primers were used: 5′-ACCCGTAGTGTTGTGGTCTG-3′ (forward), 5′-GCCATCTTCCAACTGCCTCT-3′ (reverse). The PCR results were analyzed by Southern blotting.

### Transwell assay

Transwell assays were employed to estimate the cell migration and metastasis abilities. Matrigel (BD, Franklin Lakes, NJ, USA) was diluted with serum-free medium at a ratio of 1:9 and then added to the bottom of an 8 μm transwell chamber (Corning, Kennebunk, ME, USA) and cultured in an incubator at 37 °C for 2 h. HCT116 and SW480 cells were pretreated with serum-free medium for 24 h and seeded in the upper chambers at a density of 5 × 10^4^. Serum-free medium was added to the upper chambers, and complete medium was added to the bottom chambers. After 24 h of culture under different conditions (normoxia or hypoxia), the cells were fixed with methanol and stained with a 0.1% crystal violet solution. The cells were counted under a microscope after washing with PBS. Four to six high power fields (400×) photos were taken randomly to count the number of cells in each group.

The migration assays were performed without Matrigel. SRT1720 (200 nM, Selleckchem, Houston, TX, USA) and EX527 (2 μM, Selleckchem) were used to pretreat the SW480 and HCT116 cells, respectively, in the experiment.

### Statistical analysis

All data are presented as the mean ± standard deviation (SD). *Student’s t* test was employed to compare two unpaired treatment groups. LDS-*t* test was employed for multiple comparisons. One-way ANOVA was used to analyze three or more treatment groups. ImageJ (version 1.3.7, NIH, USA) was used to measure densitometry of immunoblotting for each panel. Statistical analyses were performed by SPSS 22.0 software (SPSS, Inc., Chicago, IL, USA) and graphs were created using GraphPad Prism software (version 5.0, San Diego, CA, USA). Results demonstrating *p* < 0.05 were considered statistically significant.

## Results

### Hypoxia reduced SIRT1 expression and transcription in CRC cells

**Fig. 1:**
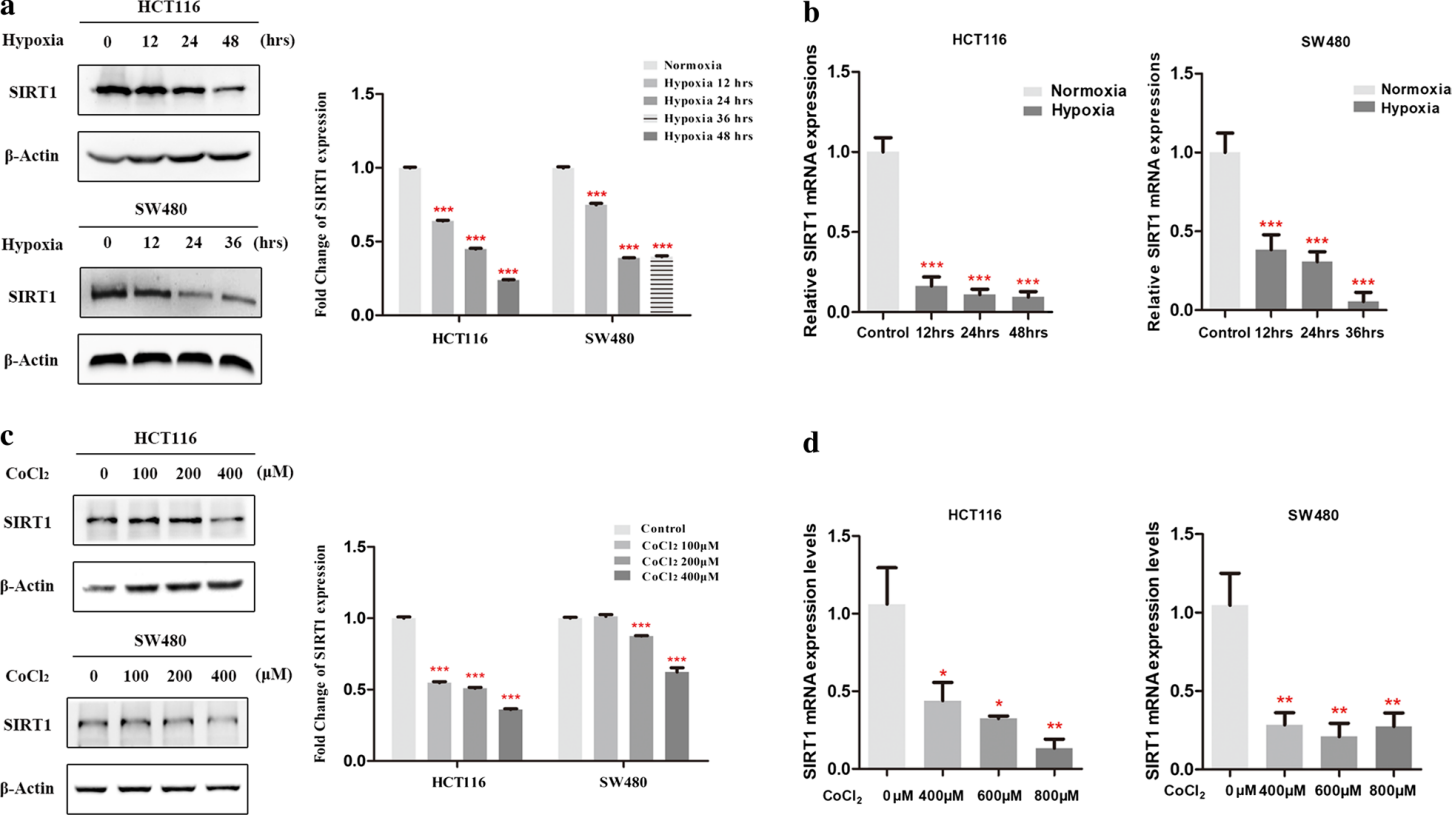
Hypoxia reduced SIRT1 expression and transcription in SW480 and HCT116 cells. **a** Western blot analyses of SIRT1 expression levels after HCT116 and SW480 cells were exposed to hypoxia. Scanning densitometry of immunoblotting for each panel was measured (right). **b** Real-time PCR analysis of SIRT1 mRNA levels after HCT116 and SW480 cells were exposed to hypoxia. **c** Western blot analysis of SIRT1 expression levels after HCT116 and SW480 cells were treated with cobalt chloride (0, 100, 200, 400 μM) for 24 h. Scanning densitometry of immunoblotting for each panel was measured (right). **d** Real-time PCR analysis of SIRT1 mRNA levels after HCT116 and SW480 cells were treated with cobalt chloride (0, 100, 200, 400 μM) for 24 h. *p < 0.05; **p < 0.01; ***p < 0.001

To determine the effects of hypoxia on CRC cells, we exposed HCT116 and SW480 cells to hypoxic conditions (1% O_2_) on a temporal gradient for up to 48 h. Then, Western blot and real-time PCR assays were performed to determine the changes in SIRT1 protein and mRNA expression levels. As indicated, hypoxia significantly reduced both SIRT1 protein and mRNA expression levels in both CRC cell lines (*p* < 0.001) (Fig. 1a, b). Cobalt chloride is a widely used chemical compound for exploring hypoxic responses in cultured cells [12]. Thus, we also employed a series of cobalt chloride concentrations to further examine the effects of hypoxia on SIRT1. Similarly, the Western blot and real-time PCR results showed that the protein and mRNA expression levels of SIRT1 were significantly decreased compared to those in the control groups (*p* < 0.001) (Fig. 1c, d). In conclusion, our results showed that hypoxia reduced SIRT1 expression in CRC cells.

### SIRT1 inhibited the hypoxia-mediated migration and invasion of CRC cells

**Fig. 2:**
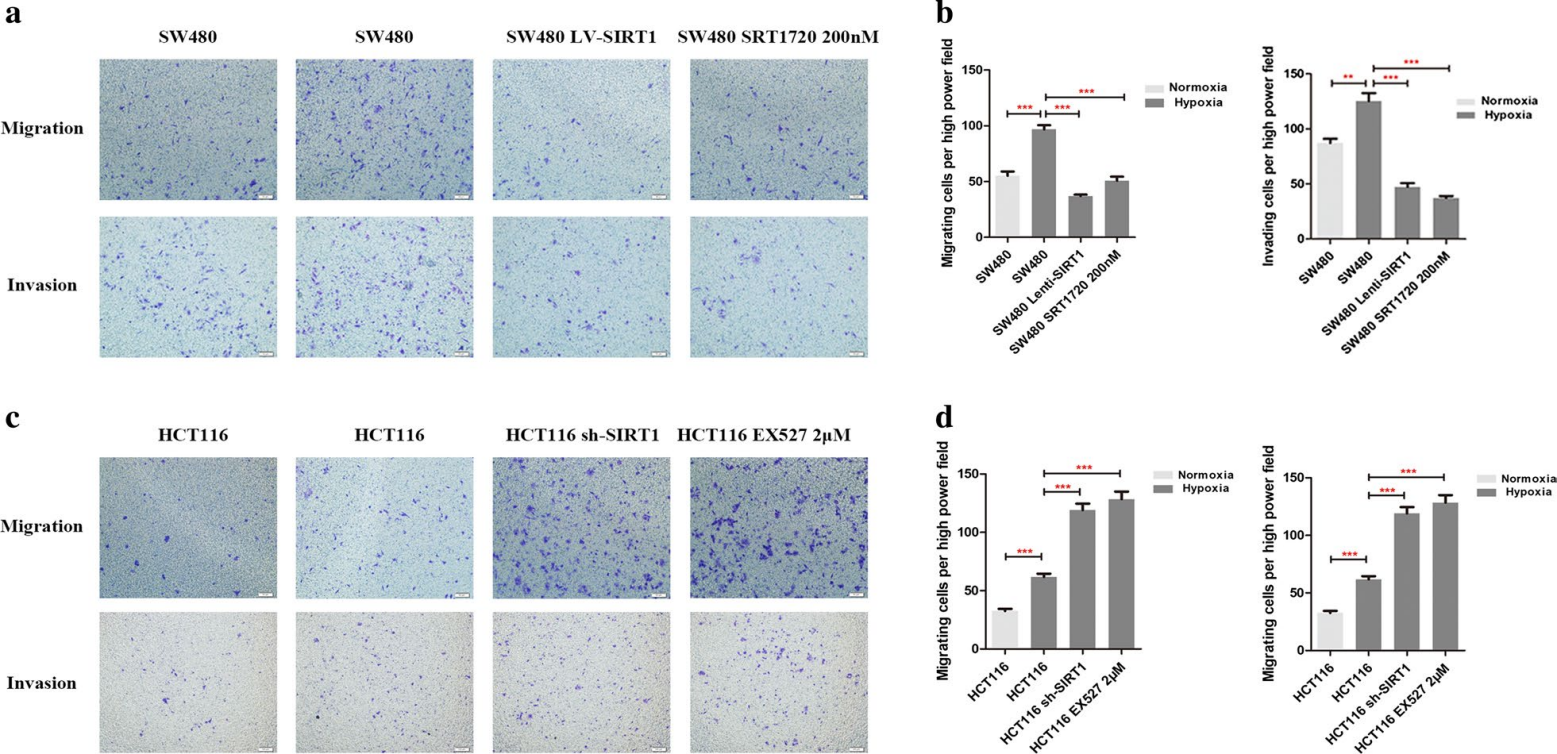
Hypoxia promoted CRC cell migration and invasion in a SIRT1-dependent manner. **a** Transwell assays were used to detect the migration and invasion of SW480 cells. SW480 cells were stably transfected with lentivirus to overexpress SIRT1 or treated with the SIRT1 activator SRT1720 (200 nM, Selleckchem). The different SW480 cell treatment groups were then subjected to normoxia or hypoxia for 24 h. **b** Column diagram indicating the migrating and invading cell counts per high power microscope field and the statistical analysis results. Five random high power fields were counted for each group. **c** Transwell assays were used to detect the migration and invasion of HCT116 cells. HCT116 cells were stably transfected with lentivirus to knockdown SIRT1 or treated with the SIRT1 inhibitor EX527 (2 μM, Selleckchem). The different HCT116 cell treatment groups were then subjected to normoxia or hypoxia for 24 h. **d** Column diagram indicating the migrating and invading cell counts per high power microscope field and the statistical analysis results. Five random high power fields were counted for each group. **p < 0.01; ***p < 0.001

A hypoxic microenvironment promotes the migration and invasion of tumor cells, and advanced tumor stages are thus observed frequently. To further test our hypothesis in CRC cells, transwell assays with or without Matrigel were used to investigate the invasion and migration properties, respectively. SRT1720 is a potent SIRT1 activator that has been shown to increase the deacetylation of SIRT1 substrates in vivo and in vitro [13, 14]. Another synthetic molecule, EX527, has been shown to be a potent inhibitor of SIRT1 [15]. Both compounds were used to modulate the activities of SIRT1. HCT116 and SW480 cells were stably transfected with lentivirus to knockdown or overexpress SIRT1, respectively (Additional file 1: Figure S1B, C). As shown in Fig. 2, hypoxia increased the migration and invasion abilities of CRC cells (*p* < 0.01). When tumor cells were treated by 200 nM SRT1720, cell migration and invasion were significantly reduced (*p* < 0.001), and these effects were recapitulated by the overexpression of SIRT1 (Fig. 2a, b). Conversely, when SIRT1 was inhibited or downregulated, the migration and invasion abilities of HCT116 cells were further increased compared with those of the untreated groups (*p* < 0.001). Collectively, hypoxia promoted CRC cell migration and invasion, and this process could be interrupted by the activation of SIRT1.

### EGR1 regulated SIRT1 transcriptional activity under hypoxic conditions

**Fig. 3:**
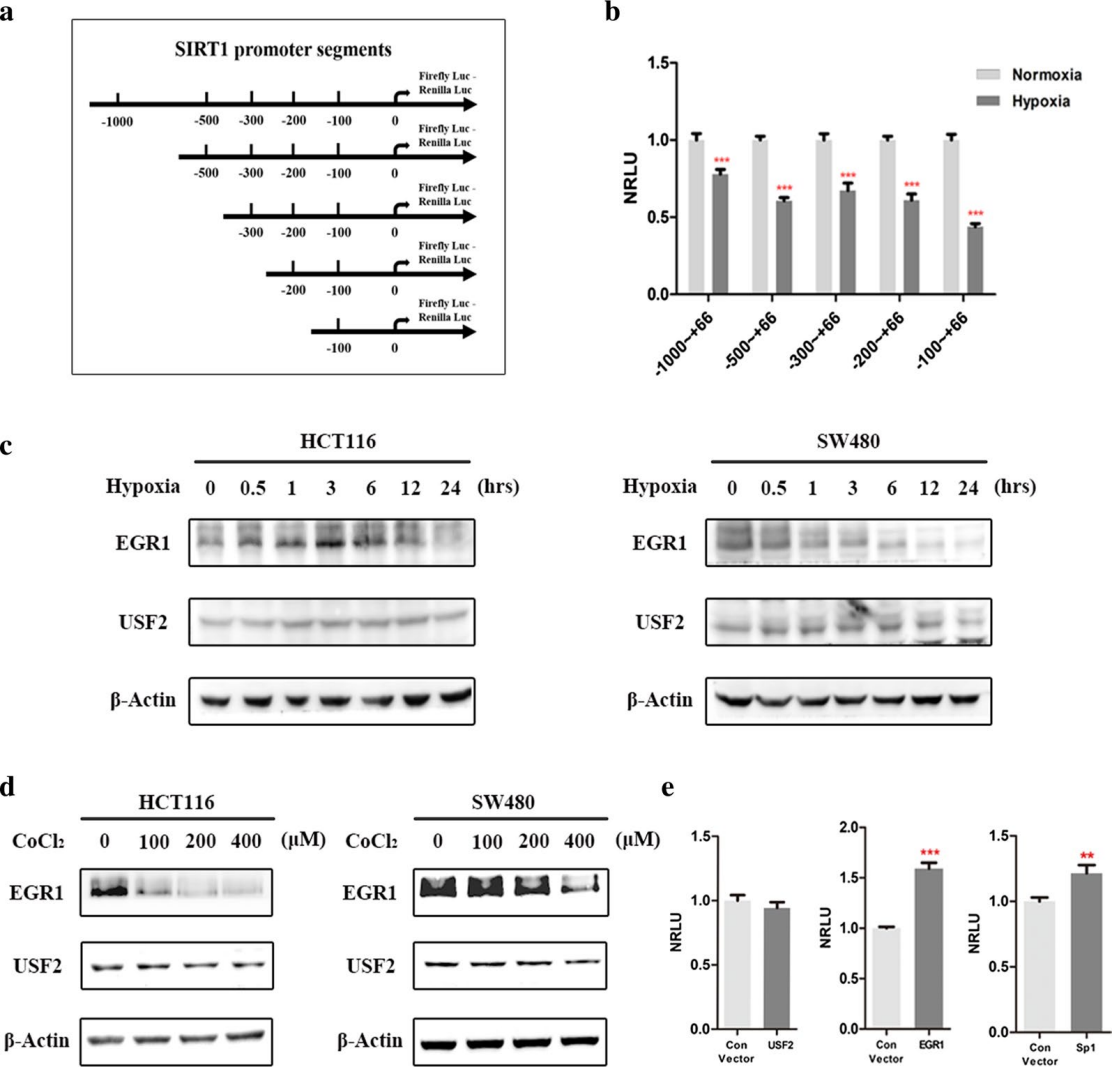
EGR1 regulated SIRT1 transcription under hypoxic conditions. **a** Schematic model indicating the construction of truncated SIRT1 promoter segments. The SIRT1 promoter sequence was truncated randomly from distal to proximal and fused with firefly and Renilla luciferase gene sequences. **b** The results of dual-luciferase reporter system demonstrated that the transcriptional activities of the different truncated segments were all inhibited by hypoxia. Each group was analyzed in triplicate, and each experiment was performed three times. **c** Western blot analysis of EGR1 and USF2 expression levels in HCT116 and SW480 cells exposed to hypoxia. **d** Western blot analysis of EGR1 and USF2 expression levels after cobalt chloride treatment (0, 100, 200, and 400 μM) for 24 h. **e** Dual-luciferase reporter system analysis of the transcriptional activities of USF2, Sp1 and EGR1 for the − 100 ~ + 66 bp segment of the SIRT1 promoter region. Each group was analyzed in triplicate, and each experiment was performed three times. **p < 0.01; ***p < 0.001

To elucidate the mechanisms through which hypoxia regulates SIRT1 transcription, we truncated the approximately 1000 bp SIRT1 promoter into five segments from distal to proximal and fused them with firefly and Renilla luciferase genes (schematic model in Fig. 3a). The SIRT1 promoter sequence was obtained from the NCBI database (http://www.ncbi.nlm.nih.gov/pubmed/). HEK-293T cells were transiently transfected with plasmids with the different truncations, and their luciferase activities were then explored using a dual-luciferase reporter gene system. Hypoxia significantly inhibited the transcriptional activities of all SIRT1 promoter constructs (*p* < 0.001), which implied that the core regulatory elements might be located 100 bp upstream of the SIRT1 promoter (Fig. 3b).

We next attempted to seek the transcription factors accounting for the transcriptional regulation of SIRT1. Bioinformatics analysis was employed to predict the potential transcription factors that are involved in SIRT1 regulation by directly binding to this section of the promoter (http://tfbind.hgc.jp/ and http://jaspar.genereg.net/). We found that Sp1, EGR1 and USF2 binding sites existed within the 100 bp upstream promoter of SIRT1. Among these, Sp1 is a transcription factor that has already been proven to bind to the SIRT promoter and positively regulate the transcriptional activity of SIRT1 [8, 16, 17]. Therefore, Sp1 was used as a positive control to indicate the trans-activation efficiencies of the other transcription factors. Given the lack of evidence regarding the trans-activation effects of EGR1 and USF2 on the SIRT1 promoter in cancer, we focused on investigating the trans-activation effects of these two transcription factors.

First, Western blotting was used to detect alterations in EGR1 and USF2 under hypoxia. With hypoxic treatment for 24 h, a changed expression of EGR1 rather than USF2 was identified (Fig. 3c). The alterations of EGR1 expression were also observed when tumor cells were treated by different gradient cobalt chloride for 24 h (Fig. 3d). To test whether EGR1 and USF2 exert trans-activation effects on SIRT1, HEK-293T cells were co-transfected with plasmids for EGR1 or USF2 overexpression and a SIRT1 truncated promoter (− 100 ~ + 66 bp segment). The dual-luciferase reporter system suggested that USF2 had no influence on the transcriptional activity of SIRT1 (*p* > 0.05), while EGR1, whose fold change was even more prominent than that of the positive control Sp1, significantly promoted the transcriptional activity of SIRT1 (*p* < 0.001) (Fig. 3e).

In summary, our results suggest that the core regulatory element of SIRT1 in hypoxic conditions is located 100 bp upstream of the sequence and that EGR1 exerts significant trans-activation effects on SIRT1 under hypoxic conditions.

### EGR1 could bind to GC-rich recognition motifs of the SIRT1 promoter sequence

**Fig. 4:**
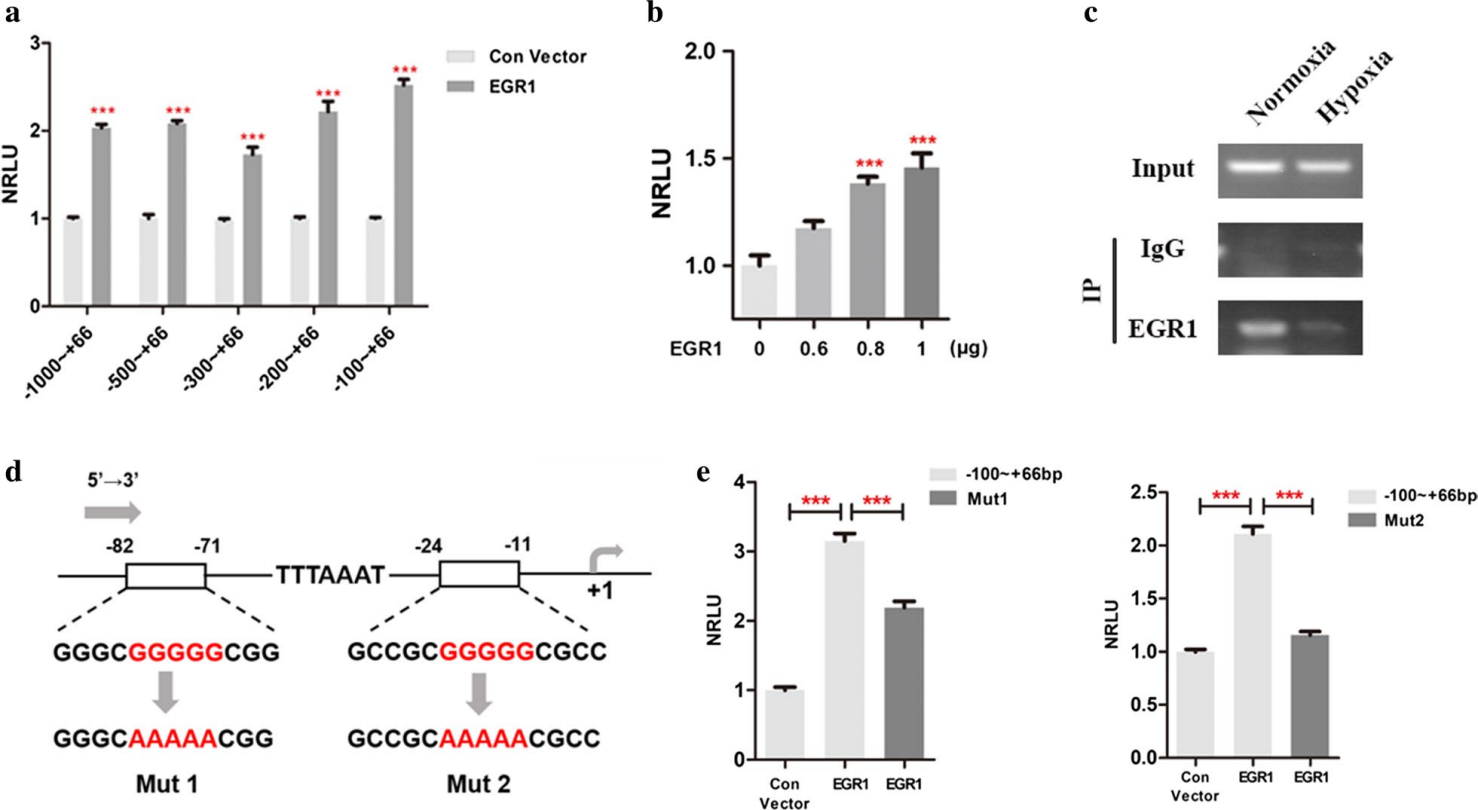
EGR1 bound directly to GC-rich recognition motifs of the SIRT1 promoter. **a** Dual-luciferase reporter system analysis of the transcriptional activities of EGR1 for all truncated segments of the SIRT1 promoter region. Each group was analyzed in triplicate, and each experiment was performed three times. **b** Dual-luciferase reporter system analysis of the transcriptional activities of increasing EGR1 concentrations for the − 100 ~ + 66 bp segments of the SIRT1 promoter region. Each group was analyzed in triplicate, and each experiment was performed three times. **c** A ChIP-PCR assay demonstrated that EGR1 binds directly to − 100 ~ + 66 bp of the SIRT1 promoter region under normoxia and disassociates from this segment under hypoxia. **d** Schematic diagram indicating the EGR1-binding GC-rich motifs and their mutated sequences within 100 bp nucleotides upstream of the SIRT1 promoter sequence. The TATA box is also indicated. **e** Dual-luciferase reporter system analysis of the transcriptional activities of EGR1 for the control and mutated promoter sequences. ***p < 0.001

To further confirm the effects of EGR1 on promoting the transcriptional activity of SIRT1, we co-transfected HEK-293T cells with EGR1 and SIRT1 constructs with truncated promoters. Consistent with previous research, EGR1 promoted the transcriptional activities of all constructs, especially the 100 bp truncated SIRT1 segments (*p* < 0.001) (Fig. 4a). In addition, the effects of trans-activation on the core regulatory element were enhanced with increasing concentrations of EGR1-overexpressing plasmids (*p* < 0.001) (Fig. 4b). A chromatin ChIP-PCR assay was designed and applied to evaluate the direct binding of EGR1 with the SIRT1 promoter. As shown in Fig. 4c, EGR1 interacted directly with − 100 ~ + 66 bp of the SIRT1 promoter when harvested under normoxia, but this interaction was dramatically hampered under hypoxia. This result suggested that under hypoxia, the expression of EGR1 was decreased, resulting in a decline in EGR1 binding to the SIRT1 promoter; thus, SIRT1 transcription was downregulated.

Next, to explore the precise binding sites of EGR1, we analyzed the 100 bp nucleotide sequence of the SIRT1 promoter and found that there are two canonical GC-rich recognition motifs (5′-GCGGGGGCG) within the − 80 ~ − 72 bp and − 21 ~ − 13 bp segments (Fig. 4d). These findings prompted us to investigate whether EGR1 bound to these motifs. Consequently, we mutated guanines in the middle to adenines and fused those with luciferase genes to construct two new plasmids (Mut1 and Mut2, Fig. 4d). These plasmids were transfected into HEK-293T cells with EGR1 plasmids. The dual-luciferase reporter system demonstrated that the trans-activation effects of EGR1 on both Mut1 and Mut2 plasmids were significantly blocked compared with those of the non-mutated plasmid (*p* < 0.001) (Fig. 4e). This experiment further suggested that EGR1 binds to GC-rich recognition motifs (5′-GCGGGGGCG-3′) within 100 bp upstream of the SIRT1 promoter.

### EGR1 reversed the migration and invasion of CRC cells under hypoxic conditions

**Fig. 5:**
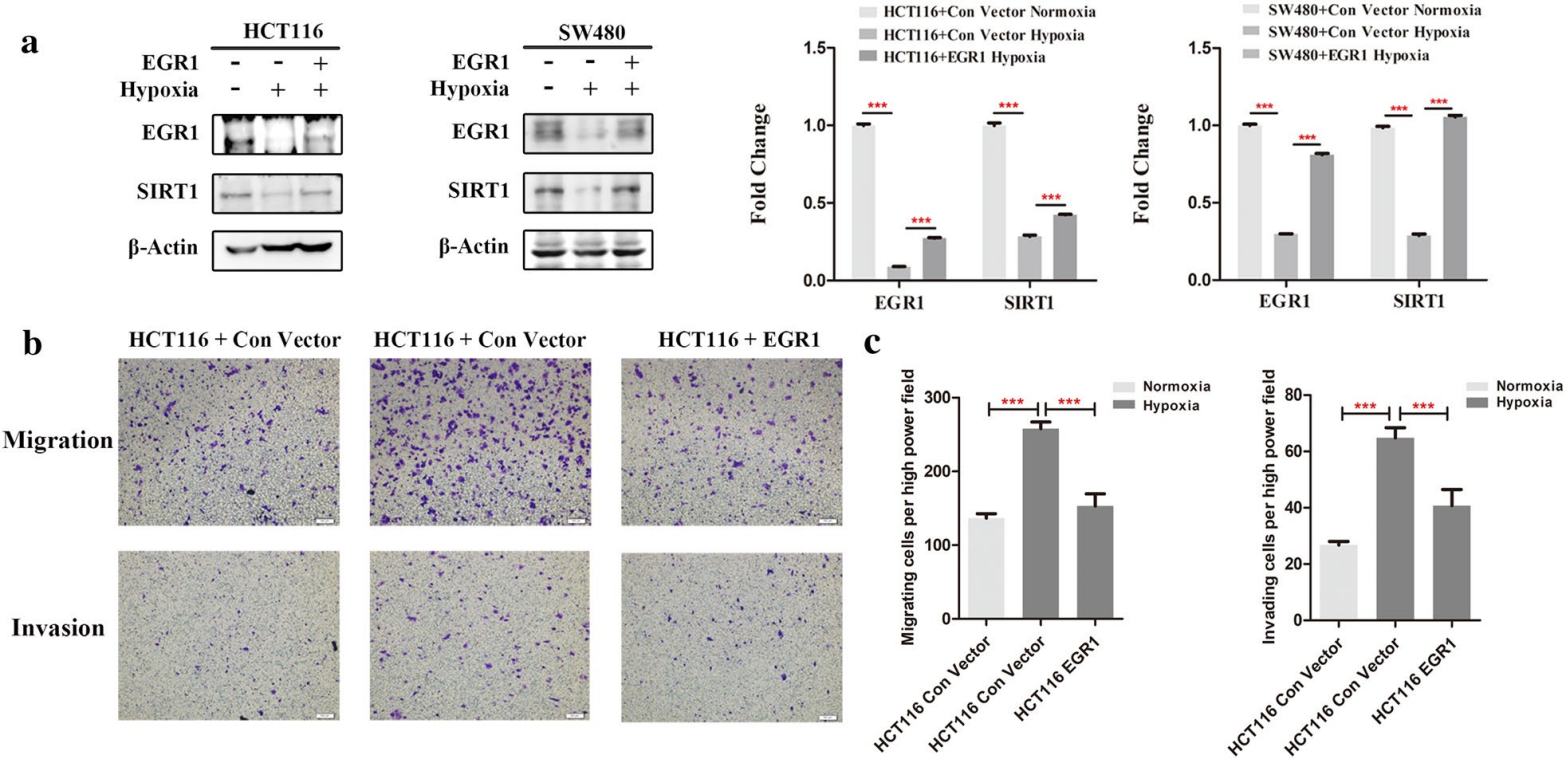
EGR1 reversed hypoxia-induced CRC cell migration and invasion. **a** An EGR1 overexpression plasmid and its empty vector were transiently transfected into HCT116 and SW480 cells, and then subjected to normoxia and hypoxia for 24 h, respectively. Cell lysates were analyzed by Western blot to detect the expression levels of EGR1 and SIRT1. Scanning densitometry of immunoblotting for each panel was measured (right). **b** An EGR1 overexpression plasmid and its empty vector were transiently transfected into HCT116 cells for 24 h. Transwell chambers were then used to detect the migration and invasion abilities of the HCT116 cells after exposure to normoxia or hypoxia for another 24 h. **c** Column diagram indicating the migrating and invading cell counts per high power microscope field and the statistical analysis results. Five random high power fields were counted for each group. ***p < 0.001

To determine whether EGR1 exerts its regulatory function under hypoxic conditions, we transiently overexpressed EGR1 in HCT116 and SW480 cells and exposed them to hypoxia for 24 h. Cell lysates were then detected by Western blot. Consistent with results above, the expression of SIRT1 was reduced under hypoxia. However, when EGR1 was overexpressed, SIRT1 expression was significantly reversed by EGR1 compared with control vector (*p* < 0.001) (Fig. 5a). Besides, the migration and invasion abilities of HCT116 cells were inhibited after EGR1 overexpression (*p* < 0.001) (Fig. 5b, c). Taken together, SIRT1 inhibition and cell motility under hypoxic conditions could be reversed by the overexpression of EGR1; that is, EGR1 plays a significant role in regulating SIRT1 in hypoxia.

### SIRT1 regulated CRC cell migration and invasion by modulating the NF-κB pathway under hypoxic conditions

In these experiments, we aimed to investigate the underlying mechanisms linking SIRT1 to the regulation of migration and invasion in hypoxia. The NF-κB pathway is a pivotal transcription factor regulating immune and inflammatory responses. It is well known that the activation of NF-κB stimulates MMPs and augments other pro-invasive markers in cancer cells [18, 19].

**Fig. 6:**
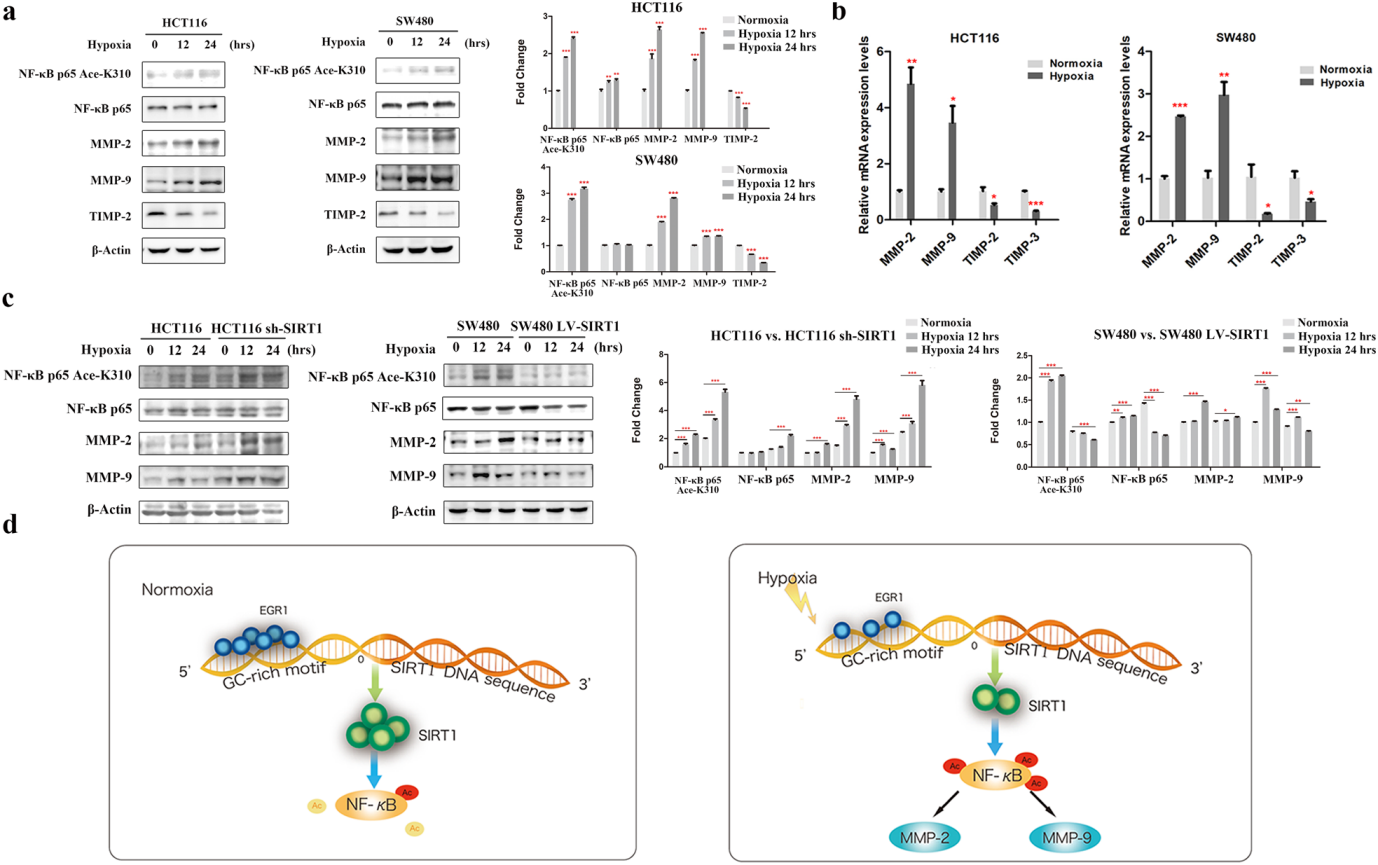
SIRT1 modulated the NF-κB pathway in CRC cells under hypoxic conditions. **a** Western blot analysis of the NF-κB p65 acetyl lysine 310 residue, NF-κB p65, MMP-2, MMP-9 and TIMP-2 in HCT116 and SW480 cells under hypoxic conditions. Scanning densitometry of immunoblotting for each panel was measured (right). **b** Real-time PCR analysis of MMP-2, MMP-9, TIMP-2 and TIMP-3 in CRC cells under hypoxic conditions. **c** Western blot analysis of NF-κB p65 and its acetyl lysine 310 residue in SIRT1 knockdown and SIRT1 overexpression CRC cells. Scanning densitometry of immunoblotting for each panel was measured (right). **d** Schematic model indicating the intracellular bioprocesses regulating SIRT1 transcription and CRC cell migration and invasion under normoxia and hypoxia

We first detected the activation of NF-κB in both CRC cell lines under hypoxic conditions. As shown in Fig. 6a, the expression level of NF-κB under hypoxia in HCT116 was slightly elevated (*p* < 0.01) while no significant change was observed in SW480 cells; however, the acetylation levels of NF-κB p65 lysine 310 residue in both cells were significantly increased (*p* < 0.05), which suggested the activation of the NF-κB pathway. Real-time PCR and Western blot assays were also employed to measure the NF-κB downstream targets MMP-2 and MMP-9. As indicated in Fig. 6a, b, the expression levels of MMP-2 and MMP-9 were increased, and their potent inhibitors tissue inhibitor of metalloproteinases 2 (TIMP-2) and TIMP-3 were simultaneously downregulated (*p* < 0.05) (Fig. 6b). In addition, compared with normoxic conditions, the acetylation of NF-κB p65 lysine 310 residue was further enhanced along with elevated MMP-2/-9 in cells under hypoxic conditions when SIRT1 was downregulated; while the acetylation of NF-κB p65 lysine 310 residue and MMP-2/-9 were reduced when SIRT1 was upregulated (Fig. 6c).

In summary, we found that SIRT1 inhibited NF-κB activation via the deacetylation of p65 lysine 310 residue. Under hypoxic conditions, SIRT1 expression was reduced. Hence, the acetylation of NF-κB was accordingly increased, which might directly and/or indirectly activate its downstream targets MMP-2/-9 and mediated CRC cell migration and invasion (The proposed schematic model shown in Fig. 6d).

## Discussion

SIRT1 is an extensively explored class III histone deacetylase and has been proven to be a trigger or brake in carcinogenesis depending on the cellular context and tumor type. In recent years, the important connection of SIRT1 to cancer metastasis has been introduced, further confirming the pivotal roles of SIRT1 in cancer progression. For example, in CRC, SIRT1 promotes EMT and metastasis in a Fra-1-dependent manner [20]. Likewise, SIRT1 is recruited to the promoter of E-cadherin and acts as a co-suppressor with ZEB1 to synergistically inhibit E-cadherin transcription, thus promoting EMT in prostate cancer [21]. Li et al. also introduced a novel mechanism whereby SIRT1 enhances PGC-1α-mediated mitochondrion biogenesis and increases cell metabolism; as a result, this process facilitates the motility of tumor cells [22]. However, some studies argue that SIRT1 suppresses cancer cell migration and invasion, such as, by targeting ARHGAP5 in gastric cancer [23], by inhibiting the TGF-β/Smad4/MMP-7 axis in breast cancer [24] and by regulating autophagy in CRC [25]. Though completely different conclusions were reached, these findings strengthen the hypothesis that the SIRT1 promotion or inhibition of cancer migration and invasion is cellular context-dependent.

Recent reports have shed light on the bi-directional interaction between hypoxia and SIRT1. On the one hand, SIRT1 inhibits the transcriptional activity of HIF-1α through the deacetylation of specific lysine sites [26]. On the other hand, the expression of SIRT1 is induced or suppressed under hypoxic conditions. SIRT1 is reportedly upregulated by HIF-1α and is involved in the promotion of cancer stem cell-like properties in ovarian cancer [9]. In contrast, SIRT1 is downregulated in non-small cell lung cancer cells exposed to hypoxia, where it modulates the chemotherapeutic resistance to cisplatin and doxorubicin [27]. Sun et al. reported that in ovarian cancer, hypoxia suppresses SIRT1 transcription via PIASy mediation of the SUMOylation of Sp1, which emphasizes the roles of SIRT1 in mediating hypoxia-induced cancer cell migration and invasion [8]. Another study from their lab observed a similar phenomenon in lung cancer [17]. This evidence prompted us to investigate the effects of the hypoxic microenvironment on the development of CRC and the relevant mechanisms.

Our data indicated that hypoxia-induced SIRT1 transcription and expression suppressed and promoted migration and invasion in CRC. Through changing SIRT1 expression and activity, we found that the bioprocess was definitely SIRT1-dependent. To determine the underlying mechanisms, we analyzed the promoter of SIRT1 and constructed a series of truncated promoter plasmids, which hinted at EGR1 as a potent transcription factor regulating SIRT1 transcription. We further verified that EGR1 binds directly to the GC-rich motifs within 100 bp of the upstream promoter of SIRT1 to significantly promote SIRT1 transcription. Under hypoxia, the expression of EGR1 was decreased, and the binding of EGR1 to the promoter of SIRT1 was decreased; as a result, SIRT1 transcription was reduced. A rescue experiment was used to further confirm the roles of EGR1 in regulating SIRT1 transcription.

EGR1 is an early-phase reactive molecule in response to various stimuli that plays a significant role in hypoxia-induced tumor progression, survival and angiogenesis [10]. Evidences show that EGR1 is a key hypoxia response factor [28]. In ischemia/reperfusion (I/R) injury, hypoxemic conditions upregulate EGR1, which is thought to be a switch in regulating inflammation, coagulation and vascular hyperpermeability [29]. In addition, a genomic profiling analysis of MCF-7 suggests that chronic hypoxia exposure increases EGR1 expression [30]. Nevertheless, after tumor cells underwent hypoxic treatment, we found that EGR1 expression levels in HCT116 cells started to increase to a peak within 3 h, then gradually declined to a lower level compared with normoxic control; whereas in SW480 cells, EGR1 expression levels continued to decline within 24 h. These results may be attributed mainly to different exposure times because almost all studies concerning alterations in EGR1 in hypoxia use acute stress models with exposure times that are relatively short (ranges from 4 to 8 h) and intermittent [29, 31]. In our study, the tumor cells were treated with a relatively long and sustained hypoxic microenvironment (24 h). Besides, the difference may be partially attributed to different cell lines that possess different responses to hypoxic stress. Hence, after treated by a long and sustained hypoxic exposure, EGR1 acts a potent regulator to influence other bioprocesses.

The NF-κB pathway is a major downstream target of SIRT1 that mediates a series of inflammatory processes in physiological and pathological conditions. Its activation induces the expression of MMPs. Our data indicated that hypoxia increased the acetylation level of the p65 lysine 310 residue, but had a minor influence on its expression. Furthermore, hypoxia promoted MMP-2 and MMP-9 and inhibited their inhibitors TIMP-2 and TIMP-3 in both CRC cell lines. The Western blot results also showed that SIRT1 overexpression eliminated the p65 acetylation induced by hypoxia along with the decreased MMP-2 and MMP-9, suggesting that NF-κB is a direct downstream target that is deacetylated by SIRT1 and might regulate CRC cell migration and invasion.

To summarize, our data emphasize the importance of SIRT1 in CRC migration and invasion under hypoxic conditions, and this regulation is EGR1 dependent. Our data establish for the first time that EGR1 functions as an important upstream regulator of SIRT1 in modulating the bioprocesses of CRC. In addition, we found that NF-κB might be an active participant in the development of CRC under hypoxic conditions. Our study imply that small molecule compounds be developed to specifically target SIRT1 to inhibit CRC migration and invasion and ultimately achieve better outcomes for patients.

## Conclusions

In conclusion, our data suggest that hypoxia promotes CRC cell migration and invasion in a SIRT1-dependent manner. EGR1 mediates hypoxia-induced SIRT1 transcriptional repression through binding to GC-rich motif of SIRT1 promoter. The potential SIRT1/NF-κB/MMP-2/-9 axis modulates cell migration and invasion in CRC.

## Additional file

**Additional file 1.** (A). Western blot analysis of SIRT1 expression levels in several CRC cells. (B). Western blot analysis of HCT116 cells stably transfected with empty and SIRT1-knockdown lentiviruses. (C). Western blot analysis of SW480 cells stably transfected with empty and SIRT1-overexpression lentiviruses.

## Abbreviations

ARHGAP5: Rho-Type GTPase-Activating Protein 5
CRC: colorectal cancer
ChIP: chromatin immune-precipitation
EGR1: early growth response 1
EMT: epithelial–mesenchymal transition
HIF-1α: hypoxia inducible factor-1α
MMP: matrix metalloproteinase
NF-κB: nuclear factor-κB
PGC-1α: peroxisome proliferator-activated receptor γ coactivator 1α
SIRT1: silencing information regulator 1
TIMP: tissue inhibitor of metalloproteinase
TGF-β: transforming growth factor-β
USF2: upstream stimulatory factor 2
ZEB1: zinc finger E-box binding homeobox 1
